# Computational genetics analysis of grey matter density in Alzheimer’s disease

**DOI:** 10.1186/1756-0381-7-17

**Published:** 2014-08-22

**Authors:** Amanda L Zieselman, Jonathan M Fisher, Ting Hu, Peter C Andrews, Casey S Greene, Li Shen, Andrew J Saykin, Jason H Moore

**Affiliations:** 1 Department of Genetics, Institute for Quantitative Biomedical Sciences, Geisel School of Medicine, Dartmouth College, Hanover, New Hampshire 03755, USA; 2 Department of Radiology and Imaging Sciences, Center for Neuroimaging and Indiana Alzheimer’s Disease Center, Indiana University School of Medicine, Indianapolis, IN 46202, USA

## Abstract

**Background:**

Alzheimer’s disease is the most common form of progressive dementia and there is currently no known cure. The cause of onset is not fully understood but genetic factors are expected to play a significant role. We present here a bioinformatics approach to the genetic analysis of grey matter density as an endophenotype for late onset Alzheimer’s disease. Our approach combines machine learning analysis of gene-gene interactions with large-scale functional genomics data for assessing biological relationships.

**Results:**

We found a statistically significant synergistic interaction among two SNPs located in the intergenic region of an olfactory gene cluster. This model did not replicate in an independent dataset. However, genes in this region have high-confidence biological relationships and are consistent with previous findings implicating sensory processes in Alzheimer’s disease.

**Conclusions:**

Previous genetic studies of Alzheimer’s disease have revealed only a small portion of the overall variability due to DNA sequence differences. Some of this missing heritability is likely due to complex gene-gene and gene-environment interactions. We have introduced here a novel bioinformatics analysis pipeline that embraces the complexity of the genetic architecture of Alzheimer’s disease while at the same time harnessing the power of functional genomics. These findings represent novel hypotheses about the genetic basis of this complex disease and provide open-access methods that others can use in their own studies.

## Findings

Alzheimer’s disease (AD) is a progressive brain disorder and the most common form of dementia. Genetic studies have revealed a number of polymorphisms associated with risk of Alzheimer’s disease. Many of these are summarized in the online AlzGene database (http://www.alzgene.org/) [1]. However, there are many additional genetic risk factors that have not been discovered using standard association methods with Alzheimer’s disease as a discrete endpoint. One approach is to use neuroimaging methods to measure brain structure and function as endophenotypes for Alzheimer’s disease. The working hypothesis is that measures of brain structure will make it easier to identify some of the undiscovered genetic risk factors for Alzheimer’s disease. The goal of the present study was to reanalyze genome-wide association study (GWAS) data from the Alzheimer’s Disease Neuroimaging Initiative (ADNI) using grey matter density as an endophenotype. More specifically, we present a bioinformatics approach that considers the joint effects of all polymorphisms and their aggregation in biologically-defined pathways. Moving beyond the standard one-polymorphism-at-a-time analysis paradigm will allow the formulation of new hypotheses about the genetic architecture of late-onset Alzheimer’s disease.

### Data

The data used in this study comes from the Alzheimer’s Disease Neuroimaging Initiative (ADNI), which began on October 1, 2004 [2]. The stated goal of this multisite study is to define the rate of progress of mild cognitive impairment and Alzheimer’s disease in order to create better treatments for these conditions. The study carried out functional magnetic resonance imaging (fMRI) every six to twelve months for 818 patients. A total of 733 with genetic data across three categories were studied here: 204 who are neurotypical, 354 with mild cognitive impairment, and 175 with Alzheimer’s disease. A total of 530,992 single-nucleotide polymorphisms (SNPs) were measured across the human genome and passes quality control as part of a previous genome-wide association study (GWAS) [3]. The combination of brain imaging and GWAS data makes it possible to carry out voxel-wise genome-wide association studies (vGWAS) creating a many-to-many mapping problem [4]. In addition to the individual voxels, there are many different phenotypes that can be extracted from the brain images. Here, we analyzed grey matter density to identify new candidate genes for Alzheimer’s disease. The details of the genotypic and phenotypic data has been previously described [3].

### A bioinformatics pipeline

**Figure 1:**
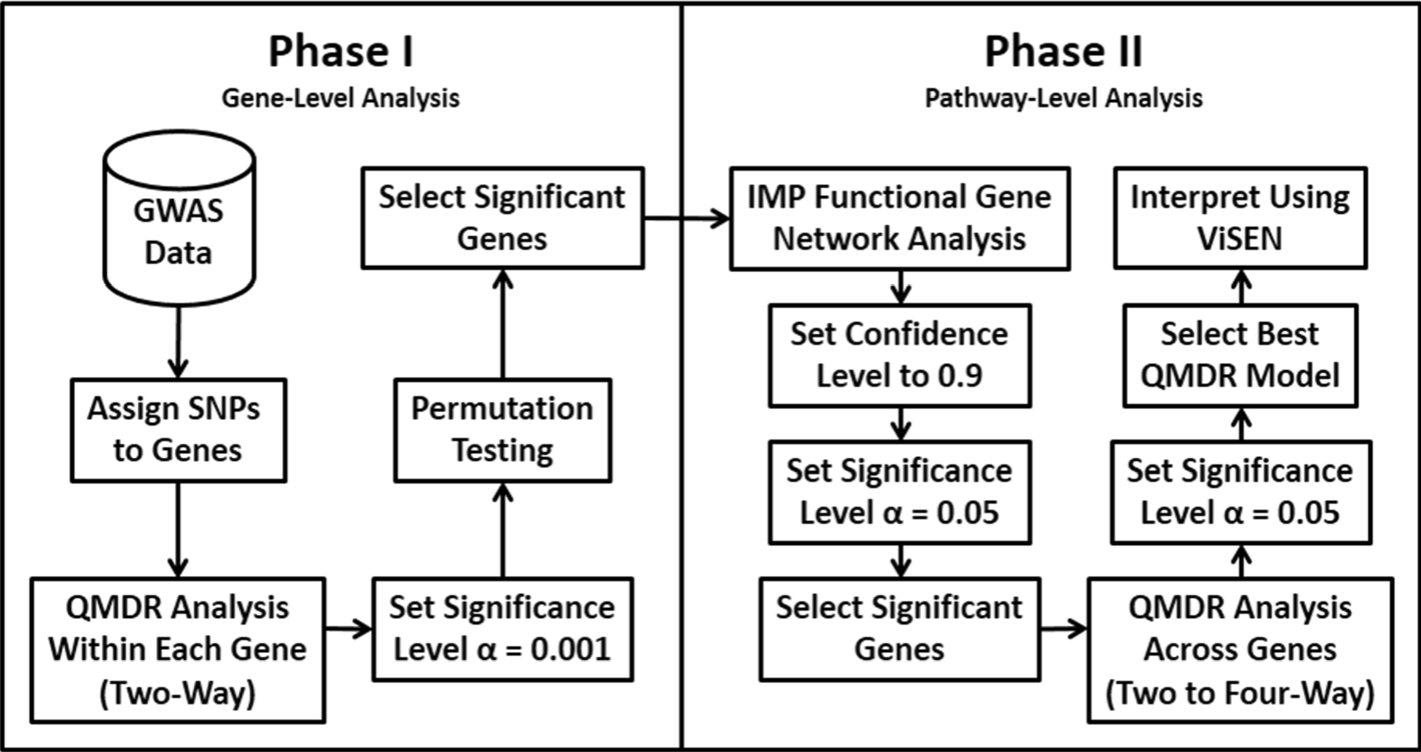
**An overview of our bioinformatics analysis pipeline.** In phase I we focus on identifying those genes with statistically significant pairs of SNPs that are associated with the phenotype. These genetic effects can be additive or non-additive for each genes. The goal of Phase II was to use bioinformatics analysis with functional genomics data to reduce the possibility of false-positive results. A final genetic model is constructed and interpreted.

The motivation for this analysis approach is to identify gene-gene interactions in Alzheimer’s disease that are not predicted by univariate effects. We combine powerful machine learning methods for detecting synergistic interactions with functional genomics data to reduce the likelihood of identifying false-positive results. Figure 1 provides an overview of our bioinformatics analysis pipeline. All methods are implemented in freely available software packages making this analysis accessible to anyone with basic bioinformatics skills. The goal of Phase I was to carry out a joint analysis of all pairs of SNPs within each gene (i.e. gene-level analysis) to allow identification of both additive effects and non-additive genetic interaction effects. The goal of Phase II of the analysis was to use a bioinformatics approach with functional genomics data (i.e. pathway-level analysis) to further address the possibility of false-positives followed by a final QMDR analysis to assess gene-gene interactions.

### Phase I: SNP-SNP interaction analysis

We first mapped each of the SNPs from the GWAS to individual genes using a window of 500 kb upstream and downstream from the gene sequence. This window size was selected to capture as many regulatory SNPs as possible without assigning any one SNP to too many different nearby genes as has been previously used for these kinds of studies [5]. Next, we carried out an exhaustive joint SNP analysis within each gene region using the Quantitative Multifactor Dimensionality Reduction (QMDR) method. MDR is a nonparametric and genetic model-free machine learning approach for detecting genetic associations that exhibit additive or non-additive effects [6]. MDR uses a constructive induction approach to map genotypes combinations from two or more SNPs to a new single variable that makes interactions easier to detect [7]. The QMDR extension allows modeling of quantitative traits such as grey matter density by collapsing multilocus genotypic means into those above and those below the global mean [8]. The means of the two new groups are then compared using a two-sample t-test. In this stage of the analysis we ran QMDR only on all pairs of SNPs within a gene region. The most informative pair of SNPs for each gene was selected and the statistical significance of their corresponding QMDR models determined using a 1000-fold permutation test. The p-value of this pair of SNPs was used to assign a p-value to its corresponding gene. We used a significance level of 0.001 to select genes for the next step of the analysis. This significance level was selected to minimize type II error (false-negatives) while providing moderate control of type I error (false-positives) due to multiple testing. At this stage of the analysis we were more concerned about type II errors than type I errors. We further address the possibility of false-positives in the Phase II analysis using functional genomics data and bioinformatics analysis.

### Phase II: functional genomics analysis

The list of genes selected in Phase I were used as input for the integrative multi-species prediction (IMP) webserver (http://imp.princeton.edu) that infers gene relationships using a Bayesian analysis of functional genomics data including thousands of publically available gene expression datasets [9]. The ultimate goal of this analysis is reduce the likelihood of including false-positive genes from the statistical analysis in phase I by focusing on those genes with strong biological evidence for biological interaction. For this analysis we used a very high confidence level of 0.9 for inferring that any two genes are functionally connected. We also allowed IMP to add up to 20 additional genes in the network that were connected to our list of genes with a high confidence. This is a standard option in the software. The output of IMP is a functional gene-gene interaction network. In addition, IMP performs a gene set enrichment analysis on the genes in the network to identify those pathways with more genes than expected by chance. We used a statistical significance level of 0.05. A final list of genes appearing in the IMP network and the significantly enriched pathways were selected for final analysis with QMDR. Here, we used QMDR to model all pairwise, three-way and four-way gene-gene interactions among the SNPs in this gene list that were identified in Phase I. Finally, we assessed the nature of the gene-gene interactions (i.e. independent, redundant or synergistic) from the QMDR results by performing entropy-based analyses [10,11] using the visualization of statistical epistasis networks (ViSEN) software package [12].

## Results and discussion

Using QMDR, phase I of the analysis revealed a total of 20 genes with 34 unique SNPs that passed the statistical significance threshold for SNP-SNP interactions (p < 0.001). The functional relationships of these 20 genes were inferred in Phase II using a bioinformatics approach that considers the correlation of gene pairs across thousands of gene expression datasets in addition to other information such as protein-protein interactions. In addition, a gene set enrichment analysis was performed to determine whether genes appearing in the functional network occurred more frequently than expected by chance in particular biological processes as defined by Gene Ontology. This latter analysis revealed enrichment for three visual perception pathways (p < 0.05) representing three genes from the gene network (CACNA1C, FKBP4, and TRPC4) as well as two pathways for DNA repair and replication (p < 0.05) representing two genes (MCM5, MCM7). An additional five genes from the olfactory pathway were present in the gene network (OR8K1, OR8K3, OR8K5, OR5R1, and OP8U1). Thus, the Phase II bioinformatics and functional genomics analysis reduced the list of 20 genes identified in Phase I to just six. Four additional genes were added in the gene network analysis based on their functional relationships for a total of 10 genes. Only 10 SNPs were present in these genes due to some SNPs mapping to more than one nearby gene. In fact, the same two SNPs represented all five olfactory genes because they are all clustered together within the 500 kb windows that were used.

An exhaustive QMDR analysis of the 10 SNPs revealed an overall best model consisting of three SNPs (rs661090, rs12222334, and rs1570612). This model was significant based on a 1000-fold permutation test (p < 0.001). The first two SNPs are located in intergenic regions within the olfactory gene cluster while the third is located in an intron of the TRPC4 gene. It is important to note that we tried and failed to replicate this finding in an independent ADNI cohort of similar size. Lack of replication could be due to different data collection methods between the two studies [13] or other factors such as differences in allele frequencies that are known to significantly impact the replication of gene-gene interactions [14].

The ViSEN analysis of these SNPs revealed that that the two olfactory SNPs had a very strong synergistic interaction in the absence of strong independent effects while the TRPC4 SNP appeared to have an effect on grey matter density that was independent of the other two (Additional file 1: Figure S1). Further, there was no evidence of correlation or linkage disequilibrium in the ViSEN analysis. Interestingly, the olfactory genes all had high-confidence functional relationships with at least one other olfactory gene suggesting that the observed synergistic effect on grey matter density might have a functional genomics basis. This is an important supporting biological piece of evidence. In addition, it is known that sensory processing, especially the sense of smell, is among the first aspects to disappear at the onset of Alzheimer’s Disease [15-20]. Interestingly, copy number variants in the olfactory gene region have been previously associated with age at onset of Alzheimer’s disease [21]. Our study is consistent with the idea that olfactory genes might play a role in the genetic architecture of Alzheimer’s disease thus making it a stronger hypothesis that needs to be further tested.

### Limitations

We presented here a bioinformatics pipeline for identifying gene-gene interactions in Alzheimer’s disease. As with any pipeline, a number of analysis decisions had to be made. For example, we selected a significance level of 0.001 in the phase I analysis and a confidence limit of 0.9 in the phase II analysis. These were selected to place more emphasis on using biological interactions to reduce false-positives due to multiple testing. Others might prefer to put emphasis on more stringent statistical criteria thus relying on statistical hypothesis testing for revealing true patterns. For example, a significance cutoff of 0.00001 in phase I would have eliminated the SNPs that were identified in our final best model. These are decisions that each user of the method will need to make based on their own experience and their own concerns about false-positives and power. In addition, it is important to qualify the p-value of less than 0.001 from the permutation testing for the final QMDR analysis since it is not entirely independent of the QMDR analyses performed in the first phase. The reader may want to take this into consideration when interpreting the final significance. Finally, although we modeled two-way and three-way interactions in this study it is possible that the genetic architecture of Alzheimer’s disease is even more complex with higher-order gene-gene and gene-environment interactions. As such, it is possible that our study is overly simplistic and that more advanced methods might be necessary.

## Availability

The MDR software package is freely available from the authors. More information can be found at http://www.epistasis.org. The IMP software is freely available at imp.preinceton.edu.

## Competing interests

The authors declare they have no competing interests.

## Authors’ contributions

ALZ and JHM developed the method, carried out the analyses, interpreted the results and drafted the manuscript. JMF, TH and PCA assisted with method development and with the programming necessary to implement the method. CSG assisted with the implementation of the IMP method and its interpretation. LS and AJS provided the data, interpreted the results and drafted the manuscript. All authors read and approved the final manuscript.

## Authors’ information

Data used in preparation of this article were obtained from the Alzheimer’s Disease Neuroimaging Initiative (ADNI) database (adni.loni.usc.edu). As such, the investigators within the ADNI contributed to the design and implementation of ADNI and/or provided data but did not participate in analysis or writing of this report. A complete listing of ADNI investigators can be found at: http://adni.loni.usc.edu/wp-content/uploads/how_to_apply/ADNI_Acknowledgement_List.pdf.

## Supplementary Material

Additional file 1: Figure S1 A SNP-SNP interaction network derived from the ViSEN analysis. Each node or vertex in the network is a SNP with a main effects proportional to the size of the circle. Lines connecting two SNPs are proportional to the size of the synergistic interaction effects after removing the one-way effects. Triangles connecting three SNPs are proportional in size to the degree of pure three-way synergistic interaction after removing the two-way and one-way effects. Note that SNPs rs661090 and rs12222334 from our final best model have a pairwise synergistic interactions with only indirect interactions with the third SNP in the model, rs1570612. Click here for file
