# Supplementary figures and images for: Computational genetics analysis of grey matter density in Alzheimer’s disease
Source: BioData Min. 2014 Aug 22;7:17. doi: 10.1186/1756-0381-7-17 (PMC4145360; doi:10.1186/1756-0381-7-17)

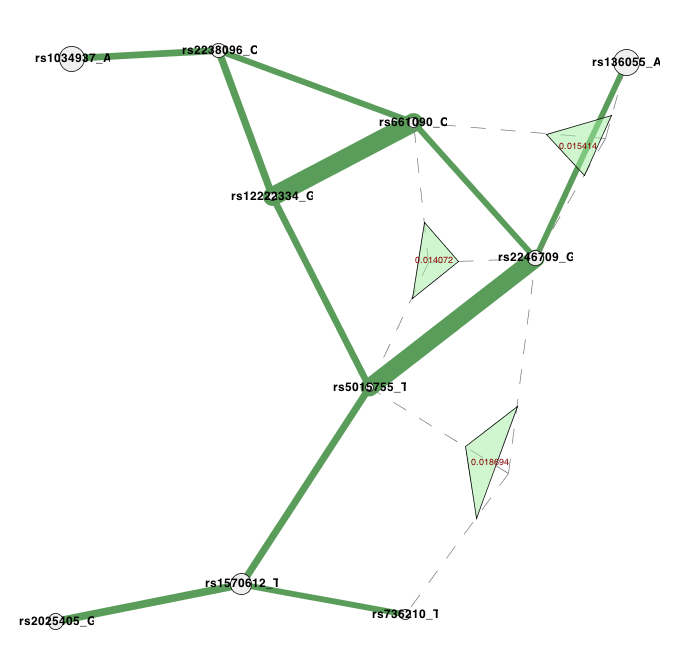

Supplement: Additional file 1: Figure S1 — A SNP-SNP interaction network derived from the ViSEN analysis. Each node or vertex in the network is a SNP with a main effects proportional to the size of the circle. Lines connecting two SNPs are proportional to the size of the synergistic interaction effects after removing the one-way effects. Triangles connecting three SNPs are proportional in size to the degree of pure three-way synergistic interaction after removing the two-way and one-way effects. Note that SNPs rs661090 and rs12222334 from our final best model have a pairwise synergistic interactions with only indirect interactions with the third SNP in the model, rs1570612. [file 1756-0381-7-17-S1.png]
